# The Effect of Wheat Germ Extract on Premenstrual Syndrome Symptoms

**Published:** 2015

**Authors:** Maryam Ataollahi, Sedigheh Amir Ali Akbari, Faraz Mojab, Hamid Alavi Majd

**Affiliations:** a *Department of Midwifery, Shahid Beheshti University of Medical Sciences, International Branch, Tehran, Iran. *; b *Department of Midwifery, Faculty of Nursing and Midwifery, Shahid Beheshti University of Medical Sciences, Tehran, Iran. *; c *Pharmaceutical Sciences Research Center, Shahid Beheshti University of Medical Sciences, Tehran, Iran. *; d *Department of Biostatistics, Faculty of Paramedicine, Shahid Beheshti University of Medical Sciences, Tehran, Iran.*

**Keywords:** Premenstrual syndrome, Wheat germ extract, Menstrual disorders, Herbal medicine

## Abstract

Pre-menstrual syndrome is one of the most common disorders in women and impairs work and social relationships. Several treatment modalities have been proposed including herbal medicines. Considering the properties of wheat germ, this study aimed to determine the effects of wheat germ extract on the symptoms of premenstrual syndrome. This triple blind clinical trial was conducted on 84 women working in hospitals affiliated to Hamadan University of Medical Sciences. Subjects completed daily symptom record form for two consecutive months. After definitive diagnosis of premenstrual syndrome, they were randomly divided into two groups of 50 people. Then, for two consecutive months, 400 mg capsules of wheat germ extract or placebo were used three times a day, from day 16 until day 5 of the next menstrual cycle. Wheat germ significantly reduced physical symptoms (63.56%), psychological symptoms (66.30%), and the general score (64.99%). Although the severity of symptoms decreased in both groups, this reduction was more significant in the wheat germ extract group (p < 0.001). On the other hand, physical symptoms decreased only in the wheat germ extract (p < 0.001) and there was no statistically significant difference in the placebo group. No complications were observed in any of the groups. It seems that using wheat germ extract reduces general, psychological and physical symptoms.

## Introduction

Premenstrual syndrome (PMS) is a common disorder in women and affects millions of young women in reproductive ages (1-4). PMS symptoms begin 7-10 days before the start of the menstrual period, and fade away in the beginning days of mense (5-7). PMS symptoms fall into two domains: psychological and physical. Its most common psychological symptoms include tension, anxiety, crying, irritability, depression, hypersensitivity, poor concentration, fatigue, mood swings and anger. Its physical symptoms include breast tenderness, abdominal cramps, bloating, swelling, acne, increased appetite, headache, backache and sleep problems (8).

The exact cause of this syndrome is unknown (6,9), but the important causes include the reaction between the neuroendocrine system, mineral and vitamin (vitamins B and D) deficiency, increased aldosterone, reduced serotonin level, genetic background and different biologic and psychological factors have received attention (1,10-13). According to studies, 75% of women with regular menstrual cycles suffer PMS (2, 6, 14-16). PMS symptoms have low and medium severity in 20-32% of women (17); while they are so severe in 3-8% of women (18, 19) that interfere with their social relationships, work and daily life (11). Not using caffeine, alcohol, egg yolk, salty foods and excessive salt and little use of foods high in fat and protein, using skim milk and low-fat milk, high-carbohydrate foods, exercising regularly, eating fruits and vegetables and getting vitamins, thiamine and riboflavin from food sources may reduce symptoms of premenstrual syndrome(7,20). Recent findings show that a change in lifestyle, exercise, not consuming caffeine, alcohol, cigarette, using minerals and vitamins such as vitamins B6 and E, calcium, magnesium, medicines such as progesterone, diuretics, spironolactone, bromocriptine, prolactin inhibitors, serotonin reuptake inhibitors, anxiolytics, beta blockers, oral contraceptives, GNRH agonists, antidepressants, NSAIDS and herbal Medicine have been effective (11,21,22). 

Herbal medicines are among the most common treatments because they are economical, safe and reliable, ease of application, non-invasive, and have fewer side-effects than do chemical medicines (23,24,25). Wheat germ, among herbal medicines, contains different kinds of vitamins, minerals and proteins, and is effective in treatment of diseases such as cancers, obesity, diabetes, asthma, anemia, eczema, hair loss, high blood pressure, ulcers and gastritis.

According to chemical analyses, wheat germ contains magnesium, zinc, calcium, selenium, sodium, potassium, phosphorus, chromium, antioxidants including beta-carotene (for vitamin A), vitamin E, vitamin C, vitamin B12, vitamin B6, thiamin, riboflavin, niacin, folic acid, iron, amino acids, and enzymes, and has a high dietary and medicinal value (26,27,28).

Various studies have been conducted on the positive effect of some wheat germ compounds (vitamins B6 and E, calcium, magnesium, essential fatty acids) on reducing PMS symptoms. Sabet Birjandi *et al.* have studied the positive effect of vitamin B6 on mitigating the physical and psychological symptoms (23). Also, Panay Moayyed studied the positive effect of vitamin B6 on reducing PMS symptoms (29). In other studies, positive effects of magnesium supplement for PMS symptoms have been addressed (30,31). Ghanbari *et al*. have pointed to the positive effect of calcium on reducing PMS symptoms (32). Furthermore, the positive effect of simultaneous intake of calcium and vitamin E on reducing more than 50% of PMS severity has been reported (33). Alexander has also confirmed the effectiveness of vitamin B6 on PMS (34). Linoleic acid has effectively reduced PMS symptoms, as well (33). 

Given the positive effects of vitamins B6 and E, calcium, magnesium, essential fatty acids for reducing PMS symptoms, and the availability of all above compounds in wheat germ, the present study was conducted to examine the impact of wheat germ extract on PMS symptoms.

## Experimental

*Method*

This triple-blind clinical trial was approved by the ethics committee of the university (reg. 116/2879), and registered in the clinical trial center (reg. IRCT201310286807N8). Then, a letter of introduction was obtained from the Shahid Beheshti University of Medical Sciences, the International Branch, and was presented to the head of the Hamedan University of Medical Sciences. 

Having acquired the license from the university, we went to all the hospitals affiliated to Hamedan University of Medical Sciences. At the first stage, 624 employed women were interviewed from whom 170 entered the study based on the following inclusion criteria: 20-45 years of age, the body mass index of 19.8-26, having no night shifts, regular menstrual periods with 21-35 days cycles and 3-10 days bleeding period, no use of antidepressants, hormones and contraceptives, and vitamins in the past 3 months. They were excluded from the research in case of relatives’ death and divorce, adverse events, pregnancy, the use of 3 or less wheat germ extract capsules in two successive days, and the use of food supplements.

The participants, then, filled out the Beck Depression Inventory (BDI). If they got score 1-10, they entered the study. The Daily Symptom Record (DSR) and demographic questionnaire were presented to them, and they were briefed about how to complete them. Research units recorded daily symptoms severity with numbers 0 (no), 1 (mild), 2 (moderate) and 3 (severe). Then, mean severity of symptoms was calculated from 1 week prior to menstrual period to 3 days after the menstrual period. The percentage of the obtained average is severity of each symptom. Total severity of symptoms headache, breast tenderness, acne, swelling, bloating and palpitations demonstrates severity of physical symptoms, and total severity of symptoms irritability, tension, sleep problems, mood swings, food cravings, wish to be alone, depression, forgetfulness, anxiety, poor concentration, crying, depression, suicide, decreased libido and fatigue shows severity of psychological symptoms. 

Also, a written consent form was taken from them. One hundred and thirty people filled out and returned the symptom daily recording form after 2 months, based on which the diagnosis of premenstrual syndrome was confirmed in 115 of them. 

Of these, 15 people refused to participate and 100 people entered the study and were divided into two equal groups using the table of random numbers; 50 in the intervention and 50 in the control groups. After identification and verification of the wheat germ powder samples in the Botanical Laboratory at the School of Pharmacy at Shahid Beheshti University of Medical Sciences, first, the extraction was performed using ethanol 96% and repeated 3 times and each time for 24 hours. The dried extracts were used to produce capsules of wheat germ extract. 400 mg capsules of wheat germ extract or placebo were used three times a day in the intervention and control groups, respectively, between the 16^th^ day of the menstrual cycle to the 5^th^ day of the next menstrual period for two consecutive months. The questionnaire’s validity was assessed through content analysis. DSR and the PMS provisional diagnosis questionnaire are standardized worldwide, and their validity and reliability have been confirmed in previous studies (14, 35, 37). 

BDI is also a global standard questionnaire whose validity and reliability have been confirmed in various studies (27, 37, 38). The data were analyzed in the SPSS-17 software. Friedman test was employed for comparison of three periods within groups, and Bonfaroni correction and the Mann Whitney test were used, respectively, pair comparisons within and between groups. 

## Results

Of the total 100 participants, 3 people were excluded for pregnancy, 3 for digestive complications, and 10 for missing or inappropriately taking the medicine. The length of the menstrual period in all participants was 3-10 days, and the interval between the periods was 21-35 days. The history of premenstrual syndrome in the wheat germ extract and in the placebo groups was 80.95% and 66.66%, respectively. On average, 73.80% of people with a positive family history of PMS experienced this syndrome. 

In order to control the confounding factors, both groups were matched before intervention for age, body mass index, income, job, education level, menarche age, marital status, family history of PMS, and there was no statistically significant difference between the two groups (Table 1). As Tables 2, 3, and 4 shows, the two groups were matched for the severity of the general, physical and psychological symptoms, and there was no statistically significant difference between them in this respect. 

**Table 1:** Demographic characteristics of the 2 groups

**Treatment Categories**		
**Variable**	**Wheat Germ Extract**	**Placebo**
Age(years) Age of menarche(years) BMI (kg/m²) education level(bachelor) marital status (single) marital status (married)	34.9 ± 6.6 13.6 ± 1.4 23.3 ± 1.8 54.7% ± 0.8 26.1±0.4 73.8±0.4	33.8 ± 6.5 13.9 ± 1.4 23 ± 1.8 61.9 ± 0.5 30.9±0.4 69±0.4

**Table 2:** Comparison of mean severity of physical& psychological &general symptoms premenstrual syndrome before and after the intervention in both groups.

**p-value**	**8 weeks after treatment**	**4 weeks after treatment**	**Before treatment**	**Treatment round groups**
physical symptoms				
p < 0.001 P=0.878	10.48 ± 7.07 24.22 ± 12.20 p < 0.001	15.48 ± 8.87 24.06 ± 14.64 p = 0.002	28.79 ± 9.08 28.29 ± 15.61 P=0.161	Wheat Germ Extract Placebo p-value
psychological symptoms				
p < 0.001 P=0.002	10.53 ± 5.41 21.72 ± 14.26 p < 0.001	15.55 ± 7.56 23.64 ± 15.83 p < 0.011	31.25 ± 13.69 29.93 ± 18.42 P=0.279	Wheat Germ Extract Placebo p-value
general symptoms				
p < 0.001 P=0.052	10.50 ± 5.12 22.97 ± 12.26 p < 0.001	15.51 ± 6.54 23.85± 14.13 p = 0.002	30.02 ± 9.88 29.11 ± 15.97 P=0.115	Wheat Germ Extract Placebo p-value

* Man-Whitney U test

** Friedman & Bonferroni

**Figure 1:**
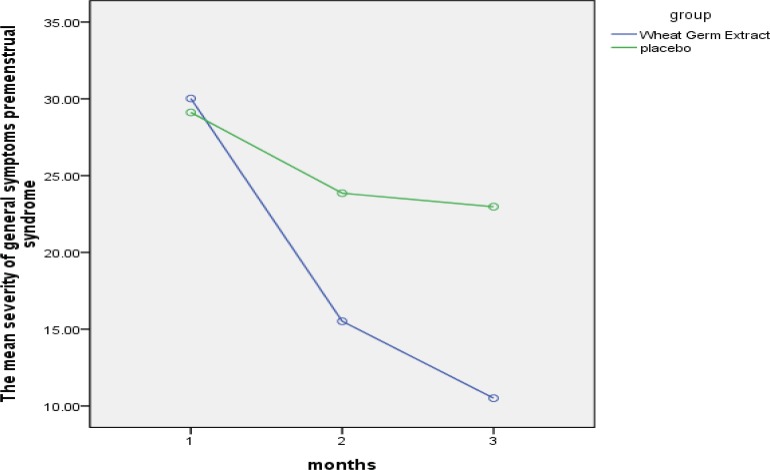
The comparison of of mean severity of general symptoms premenstrual syndrome in both groups.

The mean reduction in physical symptoms in the wheat germ extract and the placebo groups was 63.56% and 14.35%, the mean reduction in psychological symptoms in the wheat germ extract and the placebo groups was 66.30% and 27.43%: and the mean reduction in general symptoms in the wheat germ extract and the placebo groups was 64.99% and 21.05%, respectively (Table 2). The severity of the general and psychological symptoms had reduced in both groups, but this reduction was significantly greater in the wheat germ extract group (p<0.001)** (**Figure 1); while the reduction in severity of physical symptoms was statistically significant only in the wheat germ extract group (p<0.001), and there was not a statistically significant difference in the placebo group. 

The greatest reduction in severity of symptoms after two months of treatment occurred for the following in order: fatigue (85.32%), irritability (84.80%), palpitation (80.24%), tension (80.24%), breast tenderness (79.71%), headache (76.52%), sleep problems (73.79%), increased appetite (73.12%), acne (70.40%), mood swings (69.64%), food cravings (68.71%), wish to be alone (68.10%), depression (61.64%), forgetfulness (59.15%), anxiety (58.94%), poor concentration (56.72%), crying (44.53%) and swelling (25.72%).

**Figure 2:**
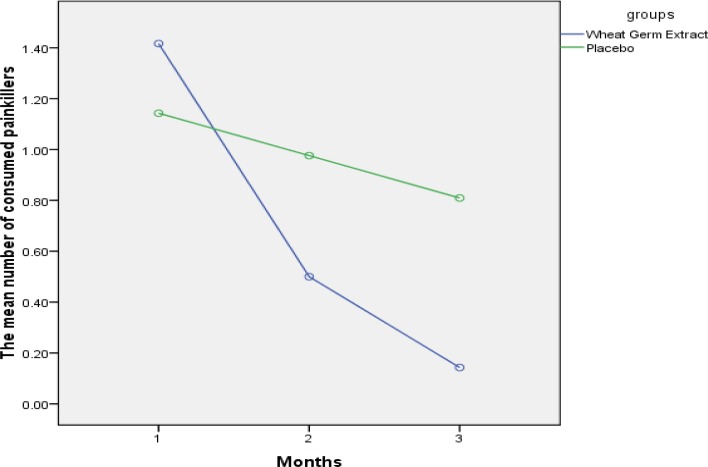
The Comparison of mean number of consumed painkillers in both groups

The Friedman test in the wheat germ extract group pointed to statistically significant differences in all symptoms (p<0.001) except the reduced desire for suicide, decreased severity of bloating and decreased libido. Neither of the groups showed changes in the duration and extent of bleeding, and there was not any statistically significant difference between the groups in this respect. The number of consumed painkillers remarkably reduced after intervention in the wheat germ extract group (p<0.001); while there was not a statistically significant difference in the placebo group on this score (Figure 2). Complications were not reported in 95.2% of the wheat germ extract group and 92.9% of the placebo group.

## Discussion

The results showed that wheat germ extract significantly reduced the severity of general, physical and psychological symptoms of PMS. The reduction in severity of general and psychological symptoms was also statistically significant in the placebo group, but this reduction was significantly higher in the wheat germ extract group, showing that the reduction in the placebo group is probably caused by the psychological effect of the medicines. The results of a study by Ozgoli *et al.,* also confirm that placebos have positive impacts on the reducing PMS symptoms (36), which is in accordance with the present study. 

There is no research directly addressing the effect of wheat germ extract on PMS symptoms. There are some notable studies on the effect of wheat germ compounds (such as calcium, magnesium, vitamins D, B6 and E). The positive effect of vitamin B6 on reducing the symptoms of depression, anxiety, irritability, breast tenderness, and the superior effect of vitamin B6 compared to placebo’s have been shown (39). Another study revealed the positive impact of vitamin B6 on all PMS symptoms (16, 41). Also, the decreased score of PMS symptoms due to supplementary zinc and vitamin B6 has been reported (35). All the three studies are in line with the present study, which might be due to the effect of vitamin B6 found in wheat germ. The positive impact of vitamin E on reducing PMS symptoms, such as anxiety and breast tenderness has been revealed, which accords with our study. This similarity is justified through the effect of the vitamin E available in wheat germ on prostaglandin synthesis and the regulation of central neurotransmitters (41). 

Although the main cause of PMS is not identified (6,9), the bulk of available evidence underlines the effect of ovarian hormones cyclic changes on central neurotransmitter mechanisms as the cause of the recurrent symptoms (4,14). Based on this, the role of B vitamins in wheat germ in regulating psychological status and mood imbalance, in particular depression in PMS, can be justified through the following mechanisms: the production of serotonin and tryptophan metabolism, pyridoxine-phosphate (the active form of the vitamin B6) in the proper synthesis of many neurotransmitters (42). The zinc in wheat germ might react with neurotransmitters which affect psychological balance (4), and reduce the symptoms. Wheat germ has sedative properties and is effective in the treatment of diseases such as ataxia, nervous system diseases and acute mental illnesses (20, 26). The iron in wheat germ is a required cofactor for the tryptophan hydroxylase. Also, the role of iron has been reported in the metabolism of serotonin and GABA. The symptoms of iron deficiency include depression, physical activity disorder, and cognitive problems, and the relationship between receiving iron and reduction in PMS symptoms has been reported (4). 

Daily intake of 1000-1200 mg of calcium has proved effective in reducing symptoms of cramp, pain, fluid retention, flatulence, appetite changes and negative attitudes (16). Also, the benefit of the daily consumption of 1200 mg calcium for reducing tension, anxiety, swelling, and the desire to eat sweets has been shown (43); all the above mentioned studies are in agreement with our research, which might be due to the impact of calcium in wheat germ. The daily intake of 1000 mg calcium with 400 IU vitamin E reduces 63% of the psychological, 55% of the physical, and 49% of the behavioral symptoms. The daily use of 500 mg calcium reduces 50% of the general symptoms in 55% of people, and 75% of the symptoms in 30% of people after three months of intervention (44), which is in accordance with our study and might be due to the effect of calcium and vitamin E in wheat germ. The positive effect of magnesium consumption for reducing fatigue severity, palpitation, headache, appetite change, swelling, chest pain, bloating, anxiety, mood swings, forgetfulness, sleep disorders, frequent crying and tension has been documented, which is compatible with our study, and might be the consequence of the positive impact of magnesium in wheat germ. The daily consumption of 45 g wheat supplement for breakfast, 40 g between the meals, and 40 g for supper reduces 75% of fatigue after two months (45), which accords with our study. Chou *et al.* have reported the positive impact of vitamin B group on reducing the severity of anxiety, mood swings, fatigue, swelling, palpitation, headache, appetite change, depression, frequent crying, forgetfulness, sleep disorders and tension, which is in line with the present research, and might be due to the effect of the vitamin B in wheat germ (46). 

The number of consumed painkillers remarkably reduced after intervention in the wheat germ extract group (p<0.001); while there was not a statistically significant difference in the placebo group on this score. The triple-blind clinical trial revealed that using 100 units of vitamin E per 6 hours for three days decreases the number of consumed painkillers (47), which is in line with our findings, perhaps because of the effect of the vitamin E of wheat germ. 

Our study did not show a significant difference between the two groups with respect to the side-effects; in fact, it is not possible to attribute any specific side-effect to wheat germ in this study. The findings of this research prove the effectiveness and safety of the daily intake of 1200 mg wheat germ extract for reducing PMS symptoms. 

Padalia *et al.,* in their review article, titled “Examining the Property of Wheat Germ Extract”, reported no side-effects in various studies conducted in this field (48), which is in accordance with our study. The examination of the effect of wheat germ extract on autoimmune diseases and cancers did not reveal any side-effects, either (49). On base of document side effects of herbal products are less than synthetic drugs (50).

## Conclusion

Consumption of wheat germ extract mitigated the severity of the general, physical, and psychological symptoms, which was observed after the first month of treatment. The therapeutic effects of wheat germ extract can be justified in view of its compounds such as vitamins B6 and E, calcium, zinc, linoleic acid, and magnesium whose effectiveness has been proved in previous studies. Wheat germ extract does not exert any effect on menstrual bleeding patterns; also, no particular side-effects were observed, and it seems that wheat germ extract can be used for controlling the premenstrual syndrome symptoms. 
